# Cultivation of Human Microvascular Endothelial Cells on Topographical Substrates to Mimic the Human Corneal Endothelium

**DOI:** 10.3390/jfb4010038

**Published:** 2013-03-21

**Authors:** Jie Shi Chua, Li Xiang Liew, Evelyn K.F. Yim

**Affiliations:** 1 T-Lab, #05-01, The Mechanobiology Institute Singapore, National University of Singapore, 5A Engineering Drive 1, 117411, Singapore; E-Mail: jieshi.chua@duke-nus.edu.sg; 2 Duke-NUS Graduate Medical School Singapore, 8 College Road, Singapore 169857; 3 Department of Bioengineering, National University of Singapore, EA-03-12, 9 Engineering Drive 1, Singapore 117576; E-Mail: liewlixiang@gmail.com; 4 Department of Surgery, National University of Singapore, Singapore 119228

**Keywords:** nanotopography, cornea endothelium, cornea regeneration, microvascular endothelial cells, contact guidance

## Abstract

Human corneal endothelial cells have a limited ability to replicate *in vivo* and *in vitro*. Allograft transplantation becomes necessary when an accident or trauma results in excessive cell loss. The reconstruction of the cornea endothelium using autologous cell sources is a promising alternative option for therapeutic or *in vitro* drug testing applications. The native corneal endothelium rests on the Descemet’s membrane, which has nanotopographies of fibers and pores. The use of synthetic topographies mimics the native environment, and it is hypothesized that this can direct the behavior and growth of human microvascular endothelial cells (HMVECs) to resemble the corneal endothelium. In this study, HMVECs are cultivated on substrates with micron and nano-scaled pillar and well topographies. Closely packed HMVEC monolayers with polygonal cells and well-developed tight junctions were formed on the topographical substrates. Sodium/potassium (Na^+^/K^+^) adenine triphosphatase (ATPase) expression was enhanced on the microwells substrate, which also promotes microvilli formation, while more hexagonal-like cells are found on the micropillars samples. The data obtained suggests that the use of optimized surface patterning, in particular, the microtopographies, can induce HMVECs to adopt a more corneal endothelium-like morphology with similar barrier and pump functions. The mechanism involved in cell contact guidance by the specific topographical features will be of interest for future studies.

## 1. Introduction

The corneal endothelium is a cell monolayer located at the cornea posterior, separating the stroma from the fluid aqueous humor of the anterior chamber. Its major function is to regulate stroma hydration, thereby maintaining corneal thickness and transparency [1]. Studies have indicated that the mature human corneal endothelial cells *in vivo* are arrested in the G1 phase of the cell cycle and, thus, do not replicate to replace dead or injured cells [2]. Instead, wound healing occurs through the enlargement and migration of adjacent healthy cells [2]. When excessive cell loss due to accidental or surgical trauma occurs, this lack of proliferative response may result in endothelial dysfunction, leading to the development of cornea edema and the eventual loss of visual acuity [2]. Current available treatments of endothelial dysfunction include penetrating keratoplasty (full thickness corneal transplantation) and endothelial keratoplasty. However, these surgical options are limited by a shortage of donor corneas and failure due to immune-mediated rejections [3]. The reconstruction of the cornea endothelium from autologous cells as a tissue engineered replacement is a promising alternative for treatment and also has potential for use as a cell model in *in vitro* ocular toxicology testing [4].

Current research focuses on the cultivation of primary human cornea endothelial cells (HCECs). The capacity of HCEC to be cultivated *in vitro* has been demonstrated, and cell layers have been cultured on temperature responsive culture dishes [5], human amniotic membranes [6] and surfaces coated with extracellular matrix (ECM) components, such as bovine corneal endothelial cell-derived ECM [7] and laminin-5 [8]. Proof-of-concept studies in animal models have shown the potential of *in vitro* cultured cornea endothelial cells for clinical use [6,9,10,11]. However, the use of these primary cells is limited by donor availability and their low proliferative capacity. It is difficult to establish a lasting HCEC culture, and aged cells tend to display abnormal morphologies [12]. Variability exists across different donors, and isolated cells can also be contaminated by stromal keratocytes, which will overtake the culture, due to their higher proliferation rate [12].

Therefore, it is necessary to consider alternative cell sources. Umbilical cord mesenchymal stem cells (UMSCs) have been transplanted in the corneas of lumican null mice with improved clarity and increased stromal thickness [13]. More recently, it has been shown that UMSCs are able to specifically attach to wounded areas of the corneal endothelium and, thus, can potentially be used for the healing of the injured corneal endothelium [14].

In this study, human microvascular endothelial cells (HMVECs) are considered and cultured as an alternative cell source for corneal endothelium replacement, because of the similarity in the microvascular and the intra-ocular pressure. *In vivo*, normal ocular pressure ranges from 10 mmHg to 20 mmHg [15], while most segments of the microcirculation have mean vascular pressures of 20 mmHg [16]. The transplantation of vascular endothelial cells as a corneal endothelium replacement in animal models has been previously reported with optimistic results, indicating their potential for use in clinical applications [17,18]. Similar to the CECs, vascular endothelial cells regulate fluid exchange. These cells form tight junctions [19] and have also been reported to possess Na^+^/K^+^ adenine triphosphatase (ATPase), which is essential for the ion transport regulation function for the corneal endothelium. However, the vascular endothelial cells in the previously mentioned studies were not characterized or tested for their resemblance to native corneal endothelium.

The basal side of the native corneal endothelium rest on the Descemet’s membrane, the basement membrane secreted by the endothelial cells. The Descemet’s membrane is a three-dimensional network of nanoscale architecture with fibers and pores [20]. Studies have shown that the use of topographies on synthetic substrates to mimic the native extracellular matrix can influence and direct cell growth and function [21,22]. The differential response of endothelial cells to various synthetic topographical structures has also been previously reported [23,24].

We hypothesized that micro- and nano-topographies of substrates can induce vascular endothelial cells to become corneal endothelial cell-like and could potentially be used for *in vitro* drug testing and the therapy of the corneal endothelium. HMVECs were grown on poly(dimethylsiloxane) (PDMS) substrates with pillar and well topographies of micro- and nano-meter sizes. The experiments were conducted in two different types of medium to test the interaction between the biochemical environment and the underlying substrate topography. The reconstructed monolayers were evaluated for cell morphology, proliferation, expression of corneal endothelial functional markers and microvilli formation.

## 2. Results and Discussion

### 2.1. Characterization of Polydimethylsiloxane Substrates Surface Pattern and Human Microvascular Endothelial Cells (HMVEC) Cultivation

**Figure 1:**
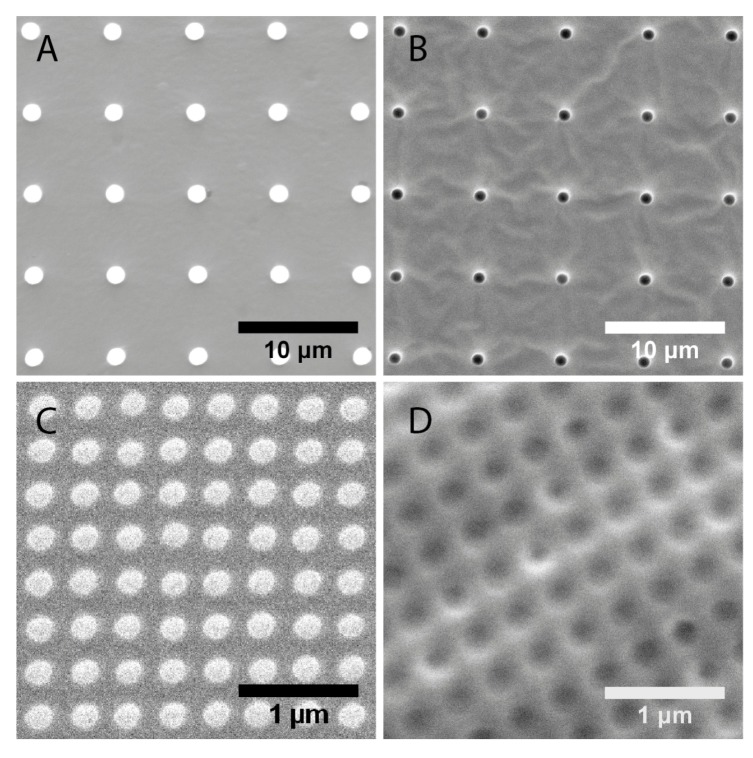
Scanning electron microscopy (SEM) characterization of substrate topographies used in this study. (**A**) 1 μm pillars; (**B**) 1 μm wells; (**C**) 200 nm pillars; (**D**) 250 nm wells.

The surface topographies of the fabricated polydimethylsiloxane substrates were examined and the structure dimensions measured with scanning electron microscopy (SEM, Figure 1). The images reflected that the fabrication technique was robust for both micro- and nano-scale structures. For the nanoscale structures and the microwells, the mean measured dimensions were within 15% of the designed dimensions (Table 1). The average diameter of the micropillars is 1.5 μm, instead of the designed 1μm. However, the value was precise, with a 1% coefficient of variation.

**Table 1:** Measured dimensions of topographies from scanning electron microscopy (SEM) images.

Topography	Designed dimensions (nm)	Measured dimensions (nm)	
	Diameter	Diameter	Pitch
1 μm pillars	1000	1500 ± 20	6810 ± 60
1 μm wells	1000	1180 ± 40	6890 ± 60
200 nm pillars	200	193 ± 11	333 ± 18
250 nm wells	250	223 ± 21	391 ± 16

The surfaces of the substrates were coated with laminin-1 to promote cell-substrate interactions. Data from a previous study showed that the laminin coat was relatively thin and did not significantly affect the surface patterns [23]. Immunofluorescence images of the laminin coating showed an even distribution over the different substrates (Supplementary Figure 1). Laminin is the most abundant non-collagenous protein found in human basement membranes and has specific binding sites, where cell surface receptors, such as integrins, can bind to [25]. The use of laminin coating in this study is to mimic the biochemical environment of the basement membranes and enhance cell attachment. Previous studies have also shown that the binding of laminin-1 to HMVECs suppresses the morphogenesis of capillary sprouts [26].

To assess the combined effects of a change in the biochemical environment and topographical cues on the HMVECs, all experiments detailed in this report, with the exception of the 5-bromo-2'-deoxyuridine (BrdU) proliferation test, were carried out separately with the endothelial cell culture medium, EGM-2MV (Lonza), and a second medium, which will be referred to as medium B throughout the rest of this report. The latter is a mixture of EGM-2MV and a medium previously reported to have been used for HCEC cultures [27,28]. The HCEC medium consists of Dulbecco’s Modified Eagle Medium (DMEM) supplemented with 15% fetal bovine serum (FBS), 1% penicillin/streptomycin and 2ng/mL basic fibroblast growth factor (bFGF, Invitrogen). For optimization, HMVECs were cultured separately in varying ratios of the HCEC medium to EGM-2MV. It was observed that the cells were unable to survive in medium consisting of 50% or higher volume percentage of the HCEC medium (Data not shown). To maintain cell viability and a reasonable proliferation rate, the final composition of medium B was decided at 75% of EGM-2MV and 25% of the HCEC medium. As HMVECs were originally adapted to Lonza’s optimized EGM-2MV, the cells could not grow well in higher proportions of the HCEC medium.

### 2.2. Formation of Tight Junctions: Zonula Occludens (ZO1) Staining

**Figure 2:**
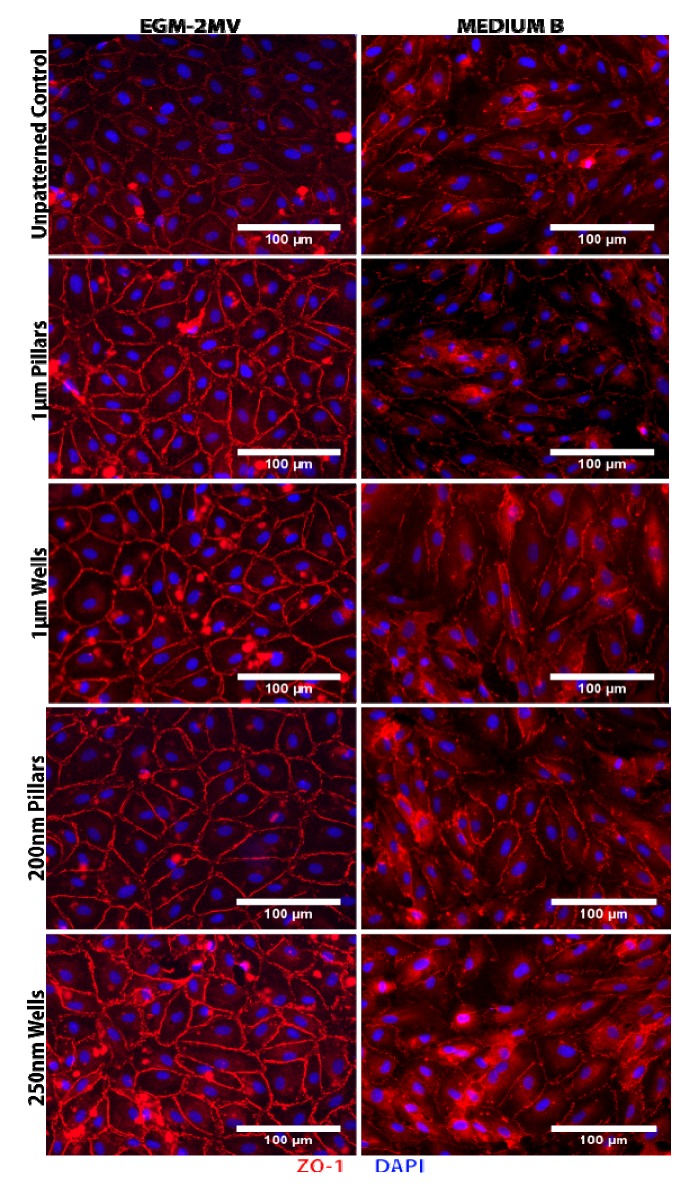
Immunofluorescence images of human microvascular endothelial cells (HMVECs) cultured on the unpatterned poly(dimethylsiloxane) (PDMS) control and PDMS with various topographies after seven days of culture in the indicated medium. The HMVECs were immuno-stained for zonula occludens 1 (ZO1, red) and DAPI (blue).

Zonula Occludens 1 (ZO1), a tight junction associated protein, was stained, and the images obtained were used to analyze cell shape and morphology. Positive staining of ZO1 was observed for all substrates grown in either medium (Figure 2). For the samples grown in EGM-2MV, ZO1 expression was localized to cell boundaries, indicating the successful formation of tight junctions. ZO1 staining was observed to be brighter on all substrates when compared to the unpatterned control, and the signal appears the brightest on nanowells (Figure 2). On the other hand, ZO1 expression on samples cultured in medium B appeared more discontinuous and diffuse (Figure 2). Intracellular junctions were less distinct, and cytoplasmic staining could be observed. There were also no detectable differences between the expression across the different topographies and the unpatterned control.

The main function of the corneal endothelium is to regulate stromal hydration through a “pump-leak” system. Fluid from the anterior chamber leaks into the stromal across low resistance apical junctions, while the activity of the ion pumps removes excess fluid and prevents stromal swelling [29]. The leaky barrier function of the endothelium is dependent on the development of tight junctions, which form a “seal” between cells [29]. The immunofluorescence ZO1 stained images of samples grown in EGM-2MV indicated the HMVEC monolayers successfully formed tight junctions and, thus, retain the barrier function of the endothelium. Stronger expression on the patterned substrates suggests that the topographical structures chosen in this study, the nanowells in particular, may aid in the maturation of the monolayers. However, tight junction formation was less obvious in the medium B samples. As the HMVECs were originally adapted for growth in EGM-2MV, it is likely that the HMVECs maturation rate is slower when cultured in medium B.

### 2.3. Cell Morphology: Polygonal Cell Shape and Cell Circularity

The ZO1 stained images were used for the analysis of cell shape and morphology. The HMVEC monolayers displayed polygonal cell shapes with the number of sides ranging from three to six (Figure 2). Native human corneal endothelium is often described as a mosaic of hexagonal cells with regular cell shape and uniform cell size (see Supplementary Figure 2B). However, morphological studies of human corneal endothelial of healthy individuals reported that while hexagonal cells are in the greatest abundance, the endothelium consists of a mixture of four- to seven-sided cells [30]. Similarly, non-uniformity had also been reported in the corneal endothelium of healthy children aged five- to 11-years-old [31]. This suggests that the heterogeneity observed in the monolayers formed may not be a reflection of poor functionality.

**Figure 3:**
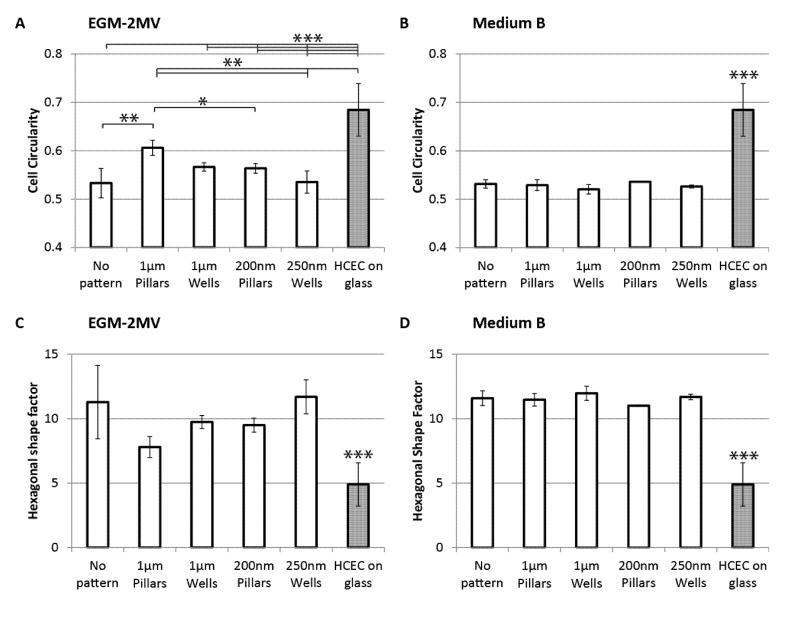
Cell circularity of HMVECs cultured on PDMS with different topographies seven days after seeding (**A**) in the medium EGM-2MV; or (**B**) in medium B; circularity = 4π × (area/perimeter^2^). A circularity value of 1.0 indicates a perfect circle. The hexagonal shape factor (HSF) of HMVECs cultured on PDMS with different topographies seven days after seeding; (**C**) in the medium EGM-2MV; or (**D**) in medium B; HSF is the absolute value of (perimeter^2^/area-13.856), where 13.856 is the shape factor of a regular hexagon. A lower value indicates less deviation from a regular hexagon shape. Circularity and HSF values of primary human corneal endothelial cells (HCECs) are analyzed from Figure 6 C and D from Peh *et al.* [32] and Figure 6 A and B from Levis *et al.* [33]. The HCECs were cultured on glass coverslips in conditions specified by the authors. Significant difference is indicated at ***, when *p <* 0.005; **, when *p <* 0.01; and *, when *p <* 0.05.

To assess the regularity and the degree of similarity of the sample cell shapes to native corneal endothelial cells, both the cell circularity and hexagonal shape factor were computed. Cell circularity is defined as 4π × (area/perimeter^2^). A perfect circle will have a circularity of 1.0, while elongated shapes have circularity values closer to 0. A regular hexagon is expected to have a high circularity value. For the monolayers cultured in EGM-2MV, it can be observed that cells grown on wells have lower circularity values and are, thus, more elongated when compared to the cells on pillars of similar dimension range (Figure 3A). The unpatterned control and the nanowells samples have similar circularity values, and the most elongated cell shape compared to the other topographies. Cells grown on micropillars have the highest circularity values. Statistically, this difference is significant when compared to the unpatterned control and the nanotopographies. On the other hand, no significant differences were observed across the samples in medium B (Figure 3B). Circularity values of cells grown in medium B are approximately 0.53, which is close to the circularity values of those grown in EGM-2MV on the unpatterned control and the nanowells.

Hexagonal shape factor (HSF) is defined as the absolute value of (perimeter^2^/area-13.856), where 13.856 is the shape factor of a regular hexagon [34]. Therefore, HSF is a measure of how much the cell deviates from a regular hexagonal shape. An ideal hexagon will have a HSF of 0, while an increase in the value indicates increasing deviation. The cell circularity results are in good agreement with the computed HSF: samples that have more circular cells also have lower HSF values, indicating stronger resemblance to HCEC. For the samples cultured in EGM-2MV, micropillars have the lowest HSF value, while the control and nanowells have the highest HSF values, although the difference is not statistically significant (Figure 3C). HSF for samples grown in medium B appears approximately similar across the different substrates with an average value of 11.5, which is close to the highest value of samples in EGM-2MV (Figure 3D).

Circularity and HSF values of primary human corneal endothelial cells (HCEC) are processed from Figure 6C and D from Peh *et al.* [32] and Figure 6A and B from Levis *et al.* [33]. It is observed that primary HCECs have significantly higher circularity and HSF than the HMVECs. The micropillars sample cultured in EGM-2MV has the closest values to the HCEC, indicating closer resemblance to the native corneal endothelium. This indicates that the topographical cues from the micropillars can induce cell shape uniformity in the HMVEC monolayer. On the other hand, the elongated cell morphologies as observed on wells could be a result of active migration. This is consistent with previous reports that cells have higher motility on substrates patterned with depressions, such as pits and pores [35,36,37]. However, HSF and circularity values of all substrates in medium B appeared similar to that of the unpatterned control in EGM-2MV. It can be postulated the topographical cues from the pillars substrates that helped regulate cell shape were masked by the change in biochemical environment when medium B is used.

### 2.4. Cell Area, Area Coefficient of Variance and Cell Proliferation

**Figure 4:**
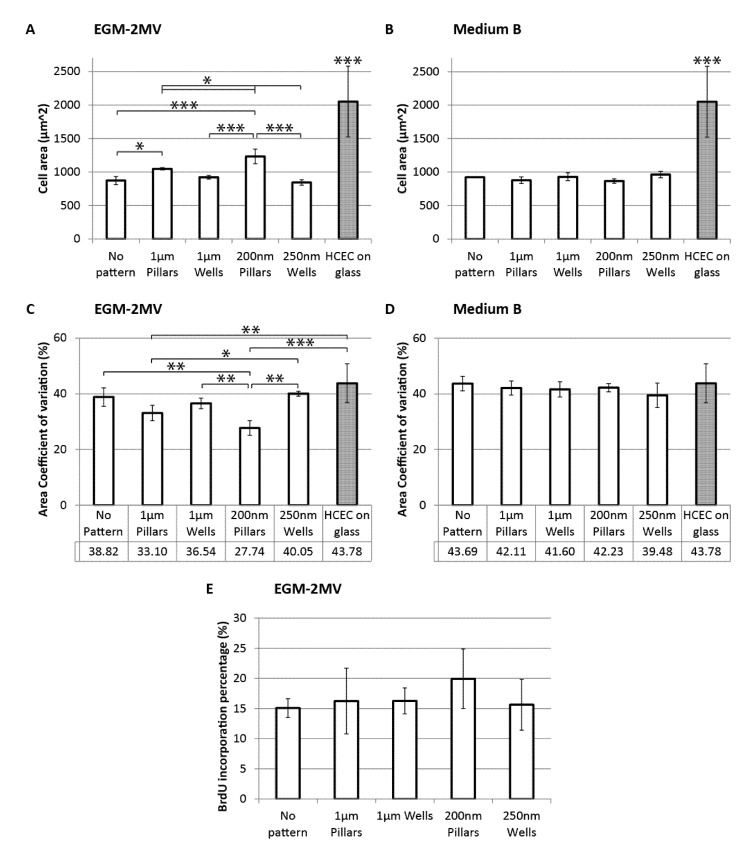
Average cell area of HMVECs cultured on PDMS with different topographies seven days after seeding (**A**) in the medium EGM-2MV; or (**B**) in medium B. The area coefficient of variance (CV) of HMVECs cultured on PDMS with different topographies seven days after seeding; (**C**) in the medium EGM-2MV; or (**D**) in medium B; (**E**) BrdU expression of HMVECs cultured PDMS with different topographies seven days after seeding in the medium EGM-2MV. Cell area and CV values of primary human corneal endothelial cells (HCECs) are analyzed from Figure 6 C and D from Peh *et al.* [32] and Figure 6 A and B from Levis *et al.* [33]. The HCECs were cultured on glass coverslip in conditions specified by the authors. Significant difference is indicated at ***, when *p <* 0.005; **, when *p <* 0.01; and *, when *p <* 0.05.

The ZO1 stained images were also used to estimate the average cell areas of the samples. For the cultures in EGM-2MV, it can be observed that HMVECs grown on pillars were significantly larger than those grown on wells of similar dimensions, while the cells on wells have similar areas to the unpatterned control (Figure 4A). Cells grown on the nanowells have the smallest cell area of 843 ± 40 µm^2^. On the other hand, the effects of topographies were not significant for the samples cultured in medium B (Figure 4B). The average cell area of the HMVECs cultured in medium B lies between 850 and 950 µm^2^, which is similar to the unpatterned control and nanowells samples cultured in EGM-2MV. Again, the effect of topography was not observable when medium B was used.

The literature reported that mean values of healthy corneal endothelium cell areas fall between 300 and 400 µm^2^. In a particular study of 58 human corneas [38], the mean cell area measured was 334 ± 51 µm^2^ (range: 273–553 µm^2^), while in a separate study of 327 cornea endothelium [39], the mean cell area was reported to be 396 ± 132 µm^2^ (range: 157–725 µm^2^). The cell areas of the HMVEC monolayers have larger values, with means above 800 µm^2 ^ (Figure 4A,B). Though the cells are considerably larger than what is to be expected *in vivo*, it does not imply a lack of resemblance to the corneal endothelium, as the *in vitro* culture of HCECs typically exhibits larger cell areas. The cell area of primary human corneal endothelial cells (HCEC) in culture were analyzed from Figure 6C,D from Peh *et al.* [32] and Figure 6A,B from Levis *et al.* [33] and measured at 2051 ± 528 µm^2^. This is considerably larger than what is expected *in vivo* and also significantly larger than all HMVEC samples.

The trend observed in the cell area values of the samples in EGM-2MV is mirrored in the BrdU proliferation assay, where samples with larger cells have higher proliferation rates. Cells on nanopillars have the highest proliferation rate, though the difference observed is not statistically significant (Figure 4E). This suggests that the topographical cues may induce cell proliferation through increased cell spreading. The correlation between the degree of cell spreading and proliferation has been previously reported in the literature [40,41,42]. Huang *et al.* [42] had shown that endothelial cells, which were prevented from spreading, were unable to enter the S-phase of the cell cycle. It is postulated that the elevated pillar topographies stabilized the formation of focal adhesions leading to stronger cell adherence, a higher degree of cell spreading and an increase in cell area, triggering the signals required for cell proliferation.

The cell area coefficient of variance (CV) was computed as the standard deviation as a percentage of the mean cell area of each sample. CV is a measure of polymegethism and is the most significant index of endothelial dysfunction [43]. For the samples cultured in EGM-2MV, CV of cells on pillars is lower than that on wells of similar dimensions and the difference is statistically significant for the nanotopographies (Figure 4C). Primary healthy HCEC typically have low CV. The mean CV of healthy adult corneas, as reported in literature, can range from 23% to 40%, depending on the age and ethnicity of the tested population [44,45,46,47]. The HMVECs monolayers on both the micro- and nano-pillars have CV values within the healthy range, as reported, while the wells topographies and unpatterned controls have higher CV values of more than 35%, more typical of older subjects. Nanopillars exhibit the lowest CV values of 27.74% ± 2.63% (Figure 4C). This suggests that topographical cues from the pillars especially in the nano range can regulate cell area uniformity. On the other hand, cells grown in medium B exhibit CV values greater than 40%, and again, no significant difference could be observed across the different substrates (Figure 4D). The cell area CV of *in vitro* primary HCEC culture was analyzed from Figure 6C and D from Peh *et al.* [32] and Figure 6A and B from Levis *et al.* [33] and measures at 43.78 ± 6.99% This value is considerably larger than what is to be expected *in vivo* and is similar to the CV values of the HMVEC samples in medium B.

### 2.5. Fluid Pump Function: Na^+^/K^+^ Adenine Triphosphatase (ATPase) Staining and the Presence of Microvilli

**Figure 5:**
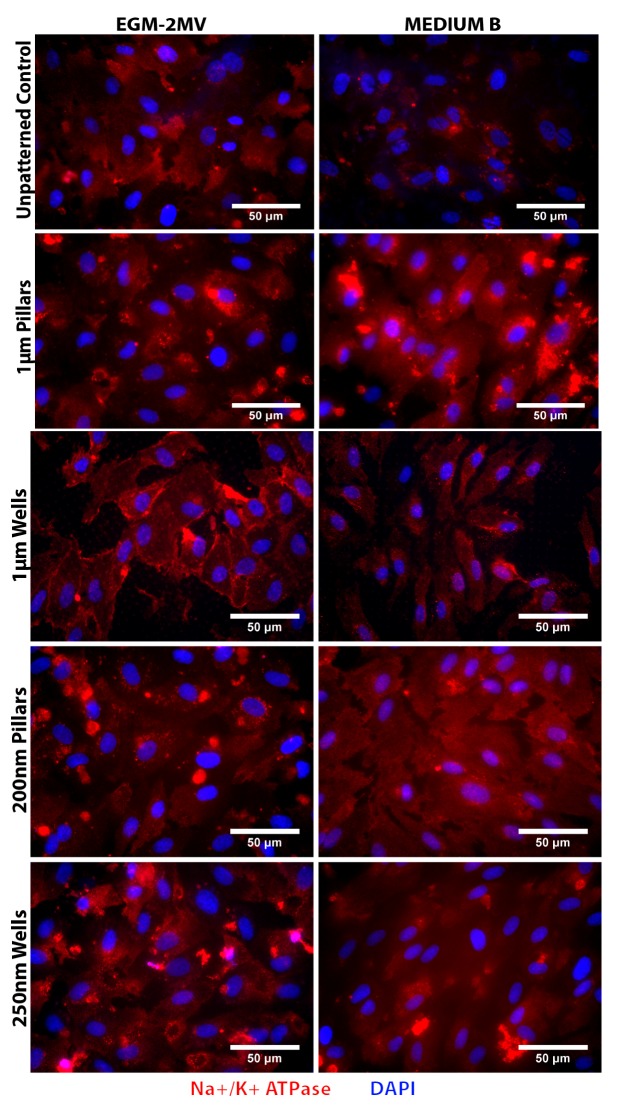
Immunofluorescence images of HMVECs cultured on the unpatterned PDMS control and PDMS with various topographies after seven days of culture. The HMVECs were immuno-stained for Na^+^/K^+ ^ ATPase (red) and DAPI (blue).

For the evaluation of the endothelial pump expression, immunofluorescence staining was used to verify the presence of the Na^+^/K^+^ Adenine Triphosphatase (ATPase). Positive staining of Na^+^/K^+^ ATPase was observed on all substrates cultured in both EGM-2MV and medium B (Figure 5). For the samples in EGM-2MV, higher fluorescence intensity can be observed for the wells topographies when compared to the pillars of similar dimensions. The Na^+^/K^+^ ATPase expression on the microwells is localized to cell boundaries, which is similar to the expression in corneal endothelium. Comparatively, the fluorescence intensity of the medium B samples appears weaker (Figure 5).

The pump function of the corneal endothelium counteracts the “leaky” barrier by removing excess fluid from the stromal. This helps to maintain the corneal transparency and prevent stromal swelling. Several ion transport pathways contributing to this function have been identified, including the Na^+^/2HCO_3_^−^ cotransporter, Na^+^/K^+^/2Cl^−^ cotransporter and Na^+^/K^+^ ATPase [48]. The preservation of the pump function in the HMVEC monolayers is demonstrated in this study by the positive expression of the Na^+^/K^+^ ATPase on all samples. In particular, the distinct staining observed at the cell boundaries on the microwells substrate in EGM-2MV suggests that the topographical cues provided by the microwells can promote the ion pump functionality of the endothelial cells.

**Figure 6:**
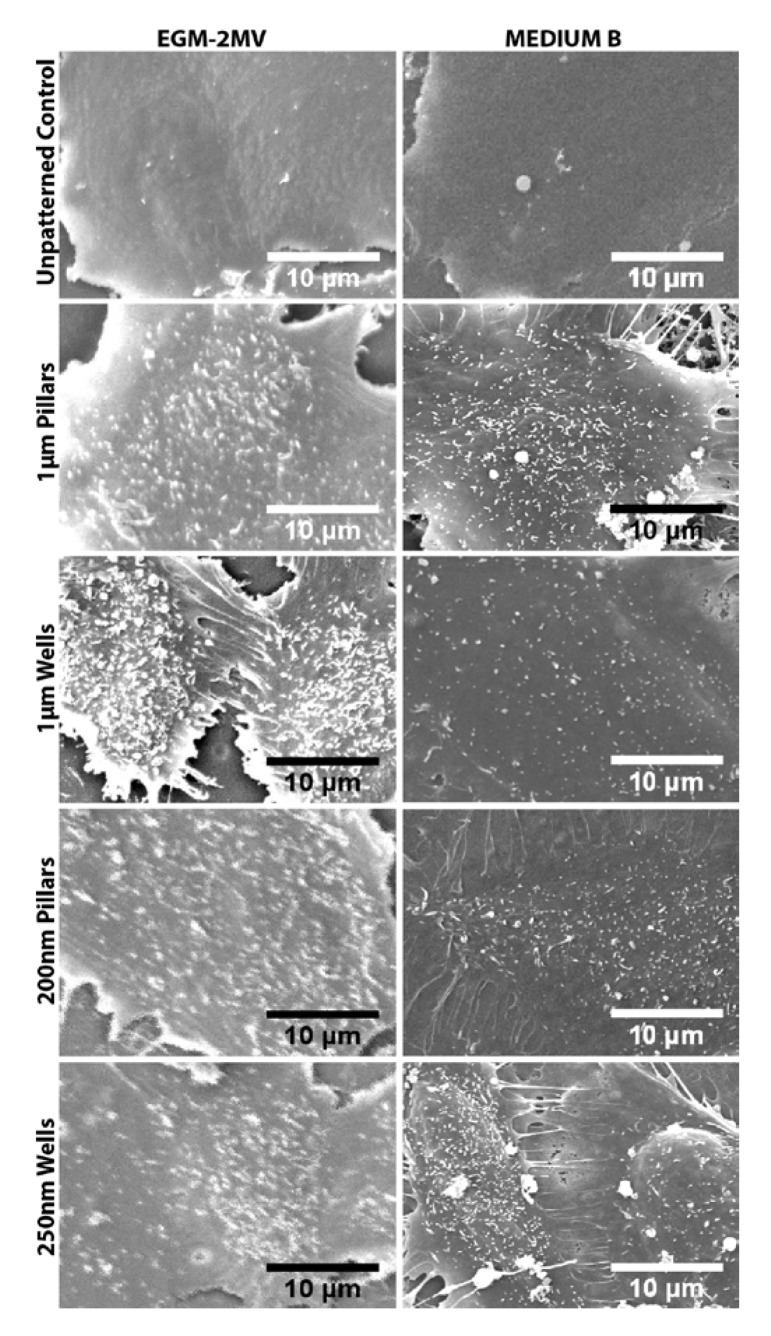
SEM of the HMVEC monolayers on the respective topographies seven days after seeding. Microvilli formation was observed on the cells of all substrates.

SEM images of the HMVEC monolayers showed the presence of microvilli on the cell surface (Figure 6). In both media, the cells on the patterned substrates had increased membrane protrusions when compared to the unpatterned control. From a qualitative observation of the SEM images, for the samples cultured in EGM-2MV, cells grown on the microwells appeared to have higher microvilli density when compared to the other substrates. The presence of microvilli and cilia on the cell surface of the corneal endothelium has been well documented in the literature [5,49]. These membrane folds increase the surface area between cells, improve cell-cell contact and help facilitate the ion pump function [1,23]. It has been reported that when the fluid pump function is blocked, there is a decrease in the microvilli number and density on the cell surface, implying that the presence of these membrane folds can be an indication of the ion transport function [50]. This suggests that the enhanced Na^+^/K^+^ ATPase expression on the microwells may be due to the higher microvilli density on the same substrate. Previous studies have also demonstrated that when cells are grown on depressed topographies, such as pits and pores, they develop a more stellate morphology with increased extensions at the edges of the depressions [35,36]. It is likely that the same discontinuity perceived by the cells on microwells substrate in this study induces increased membrane folds on the cell surface, leading to a larger surface area, which facilitates the endothelial pump function. It was also suggested in the literature that cells are able to sense the curvature of the substrate discontinuity at the depressions and the edges act as “footholds” for cell adhesion [37]. This suggests that the dimensional difference in edge geometry may have resulted in the nanowells samples not having the same degree of microvilli formation as the microwells.

For the samples grown in medium B, higher microvilli density could be observed on all the patterned substrates in comparison to the unpatterned control (Figure 6). However, less difference could be observed among the different topographies. Although the microvilli are still present, they appear less dense on the microwell samples cultivated in medium B, as compared to the same topography in EGM-2MV. It appears that while the presence of substrate topography could help promote the formation of microvilli when grown in medium B, it is to a much smaller extent.

### 2.6. Data Summary

In this study, microvascular endothelial cells are evaluated for their potential to be an alternative cell source for *in vitro* drug testing or corneal endothelium therapy. In long term cultures and *in vivo*, HMVEC exhibit contact-inhibited growth [51]. However, when stimulated, for example, during injury, these cells have the potential to proliferate, migrate and form new blood vessels [51]. On the other hand, the cornea is an avascular tissue [1], and angiogenesis is not a desirable phenomenon. However, despite their known angiogenic potential, the clinical potential of vascular endothelial cells for cornea therapy has been demonstrated in *in vivo* animal studies. Luo *et al.* [17] had shown that the injected vascular endothelial cells to the rabbit cornea were well adhered and improved clarity. In addition, the laminin coating used was previously shown to suppress the morphogenesis of capillary sprouts [26]. Nevertheless, due to the angiogenic and proliferative potential of HMVECs, it is plausible that complications may arise from the use of these cells in the cornea. Further *in vivo* studies need to be carried out to properly determine their therapeutic efficacy.

The data obtained suggest that the biochemical cues from the culture medium plays an important role in determining the cell characteristics of the HMVECs monolayers. Two-way ANOVA tests also show significant interaction effects between the topography and the medium used for all the quantifiable data (Table 2). When EGM-2MV was replaced with medium B, the differences across the patterned topographies and the unpatterned control became non-significant. This suggests that the change of the biochemical environment masked the topographical cues from the underlying substrate. For the quantifiable data, namely cell area, cell circularity, hexagonal shape factor (HSF) and area coefficient of variance (CV), the topographies are scored according to their likeness to primary HCEC, relative to the unpatterned control in EGM-2MV. Topographies with values that have a closer resemblance to the native corneal endothelium are given positive scores, while those with less desirable features are scored negatively, taking into consideration the statistical significance of the data values (Table 3). In addition, topographies are also scored for expression of ZO-1, Na^+^/K^+^ ATPase and microvilli formation from the immunofluorescence staining and SEM images. A higher score indicates stronger resemblance to the corneal endothelium. In terms of resemblance to corneal endothelium, the properties of the monolayers cultured in medium B also appeared less desirable than that in EGM-2MV and scored mostly negative in the summary table (Table 3). As the HMVECs were originally adapted and optimized for growth in EGM-2MV, it is likely that the cells could not grow well in medium B, retarding their maturation rate and resulting in more abnormal morphology. This is evident from the weak expression of ZO1 and Na^+^/K^+ ^ ATPase and the abnormally long cell processes observed in the SEM images.

**Table 2:** *p*-value of two-way ANOVA test (with Bonferroni post-test correction) of the effect of medium and topography on cell area, cell circularity and cell area coefficient of variance (CV).

Variable	Cell circularity	Hexagonal shape factor	Cell area	Cell area coefficient of variation
Medium	<0.0001***	0.0013***	0.003***	<0.0001***
Topography	0.0162*	0.0758	0.0006***	0.0171*
Interaction	0.0031***	0.0428*	<0.0001***	0.007**

Statistical significance is indicated at ***, when *p <* 0.005; **, when *p <* 0.01; and *, when *p <* 0.05.

**Table 3:** Summary: The effect of topography on the resemblance of the HMVEC monolayers to native corneal endothelium; for the quantifiable data, namely cell area, cell circularity, hexagonal shape factor (HSF) and area coefficient of variance (CV), + is given for topographies with values that have a closer resemblance to the native corneal endothelium and − is given when values appear less desirable relative to the unpatterned control in EGM-2MV. A double symbol is given for the topographies with the most or least desirable values.

Topography	Medium	Cell area	Cell circularity	HSF	CV	ZO-1	Na^+^/K^+^ ATPase	SEM	Scoring
1 µm Pillars	EGM-2MV	−*	++**	++	+	+	+	+	8
	Medium B	−	−	−	−	−	+	+	−3
1 µm Wells	EGM-2MV	−	+	+	+	+	++	+	6
	Medium B	−	−	−	−	−	+	+	−3
200 nm Pillars	EGM-2MV	−***	+	+	++**	+	+	+	5
	Medium B	+	+	+	−	−	+	+	3
200 nm Wells	EGM-2MV	++	+	−	−	+	+	+	4
	Medium B	−	−	−	−	−	+	+	−3

Statistical significance relative to the unpatterned control is indicated at ***, when *p <* 0.005; **, when *p <* 0.01; and *, when *p <* 0.05. For ZO-1 expression, + is given for patterns with well-defined tight junction staining; and − for patterns with discontinuous and diffuse staining. For Na+/K+ ATPase expression, + is given for positive staining; while ++ is given for topographies with the desired staining pattern; for the SEM images; + is given for the presence of microvilli. Each topography is given a score according to the symbols given, where + gives +1, − gives −1, * beside + gives +1 and * beside − gives −1. A higher score indicates stronger resemblance to the corneal endothelium.

For the cells grown in EGM-2MV, the topographies have differential influence in directing the growth of HMVECs (Table 3). This supports the hypothesis that surface topographies could be used as biophysical cues to direct the growth of the HMVECs. In terms of overall resemblance to corneal endothelium, the microtopographies scored higher than the nanotopographies, with the micropillars having the highest overall score (Table 3). Cells grown on the pillars were rounder and more hexagonal-like as compared to wells of similar dimensions. The HMVECs on the micropillars appears to have cell shapes that bear the closest resemblance to HCECs. On the other hand, the microwells sample had enhanced Na^+^/K^+^ ATPase expression and increased microvilli number and density, suggesting that the topography can help retain the ion pump functionality of the cells. For all topographies, ZO1 expression was localized to the cell borders and stronger than the unpatterned control, suggesting that the biophysical cues from the underlying patterns can aid the formation of tight junctions and retain the barrier function.

## 3. Experimental Section

### 3.1. Preparation of Polydimethylsiloxane Substrates

Soft lithography was used to fabricate polydimethylsiloxane substrates with micro- and nano-topographies as previously described [23]. Patterned master molds were commercially purchased in silicon wafer format. Briefly, poly(methyl methacrylate) (PMMA) (Microresist, MW 35000 g/mol) was first spin-coated on a clean silicon substrate to form a thin PMMA film. The purchased master mold was placed on top of the spin-coated surface, and the imprinting was carried out at 150 °C under a pressure of 60 bar for 10 min. Subsequently, the system was cooled before demolding the silicon master from the imprinted PMMA polymer layer. The PMMA mold was then used for soft lithography. The master molds were cleaned with nitrogen gas and fluorinated with (tridecafluoro-1,1,2,2-tetrahydrooctyl)-1-trichlorosilane (United Chemical Technologies, Pennsylvania, USA). The molds were then washed with 0.01% Triton X (Biorad, Singapore) and blown dry with nitrogen gas. PDMS base and curing agent (Sylgard 184 Silicone Elastomer Kit, Dow Corning, Singapore) were mixed with a 10:1 ratio and degassed in a desiccator for 30 min. The mixture was poured over the PDMS molds, degassed in a desiccator for another 2 h and cured at 60 °C for 12 h. Upon cooling to room temperature, the PDMS substrates were gently peeled off from the master molds.

To verify the surface topographies and to ensure the fidelity of the replication process, the PDMS substrates were sputter coated with gold (JEOL, Japan, JFC Fine Gold Coater) and examined with a scanning electron microscope (SEM, FEI, Japan, Quanta FEG 200 and JEOL, Japan, JSM-5600LV Scanning Microscope, Japan).

Prior to cell seeding, the PDMS were air plasma treated with low radio frequency (RF) power for 15 sec (Harrick Scientific Corporation, New York, USA, PDC-002), cleaned with 70% ethanol and sterilized under UV for 30 min. The substrates were also precoated with 10µg/mL of laminin (Invitrogen, Singapore) overnight.

### 3.2. Vascular Endothelial Cell Culture on PDMS Substrates

Human neonatal dermal microvascular endothelial cells (HMVEC, Lonza, Singapore) were expanded in endothelial cell growth medium, EGM-2MV (Lonza, Singapore) in standard tissue culture flasks. HMVEC of passage 7 to 11 were used for experimentation after their morphology was inspected (Supplementary Figure 2A).

All experiments detailed in this report, with the exception of the BrdU proliferation assay, were carried out in two different types of medium separately. The first is the endothelial cell culture medium EGM-2MV (Lonza, Singapore), while the second consists of 75% of EGM-2MV and 25% of medium, which had been used for the culture if HCEC primary cells [27,28]. The HCEC medium was composed of Dulbecco’s Modified Eagle Medium (DMEM, Biological Industries, Bio-Rev Singapore) supplemented with 15% fetal bovine serum (FBS, Gibco, Singapore), 1% penicillin/streptomycin (Gibco, Singapore) and 2ng/mL basic fibroblast growth factor (bFGF, Invitrogen, Singapore). The cells were seeded on the pre-prepared plasma-treated patterned PDMS substrates and unpatterned PDMS controls at densities of 2500 cells/cm^2^ for the BrdU cell proliferation assay in EGM-2MV, 40,000 cells/cm^2^ for other experiments in EGM-2MV and 45,000 cells/cm^2^ for other experiments in medium B. The HMVECs proliferate slower when grown in medium B. Therefore, a higher seeding density was used for experiments in medium B to ensure that the cells were exposed to the substrates for the same culture period. The cells were suspended in 1 mL of culture medium in 24 well plates and incubated at 37 °C and 5% CO_2_. The medium was changed on alternate days, and each experiment was ended and analyzed after 7 days.

### 3.3. Immunofluorescence Staining of ZO-1 and Na^+^/K^+^-ATPase, BrdU Proliferation Assay

The samples were fixed and stained following standard immunofluorescence staining protocol after 7 days of culture. Generally, the cells were fixed with 4% paraformaldehyde (Sigma Aldrich, Singapore) and permeabilized with 0.1% Triton X-100. They were subsequently blocked with 1% bovine serum albumin (BSA) and 10% goat serum for 1 hour at room temperature. Then, the samples were incubated with primary antibodies at 4 °C overnight, followed by an hour incubation with the secondary antibody, anti-mouse IgG Alexa Fluor 546 (Invitrogen, Singapore) diluted 1:750 at room temperature. The cells were labeled for ZO-1 or Na^+^/K^+^-ATPase, while the nucleus was counterstained with 4’,6-diamidino-2-phenylindole (DAPI). All samples were mounted onto coverslips using ProLong Gold Antifade mounting medium (Invitrogen, Singapore). The stained samples were viewed with an epifluorescence microscope (Leica, Singapore, DM IRB) and analyzed using ImageJ (National Institute of Health, Bethesda, MD, USA).

The primary antibody used for ZO-1 staining was the Mouse IgG Anti-ZO-1 antibody (ZYMED Laboratories, Invitrogen, Singapore), diluted 1:30. The images obtained from ZO-1 staining were used for the measurement of cell area, cell area coefficient of variance (CV), cell circularity and the hexagonal shape factor (HSF). CV of the cell area is defined as the ratio of standard variation to the mean and is presented as a percentage. Cell circularity is defined as 4π × (area/perimeter^2^). A perfect circle will return a value of 1.0. As the number approaches 0.0, the shape becomes increasingly elongated. HSF is defined as the absolute value of (perimeter^2^/area-13.856), where 13.856 is the shape factor of a regular hexagon. For each pattern, at least 250 cells were analyzed for each replica and each pattern type has 3 replicas.

Samples that were stained for the Na^+^/K^+^-ATPase pump followed the standard protocol, as described previously, but without the permeabilization step. The primary antibody used is the mouse IgG anti-Na^+^/K^+^-ATPase (Santa Cruz Biotechnology, Texas, USA) diluted 1:40. Random areas of each sample were photographed for analysis.

For cell proliferation studies, the cells were seeded at a low density to reduce cell-cell contact, which could inhibit cell proliferative ability. At the termination of the experiment, the samples were incubated with BrdU labeling reagent (Sigma Aldrich, Singapore) for 4 h before fixing with 4% paraformaldehyde and permeabilized with 0.1% Triton X-100 for 15 min. Next, incubation in 4N hydrochloric acid was carried out for 10 min at room temperature. The samples were stained according to the previously described standard immunofluorescence staining protocol. The primary antibody used is the mouse anti-BrdU (Developmental Studies Hybridoma Bank, Iowa, USA) diluted 1:20,000. Images of random regions of each sample were photographed and analyzed with ImageJ to obtain the number of BrdU incorporated nuclei and the total number of nuclei. BrdU incorporation percentage was then calculated as the percentage of BrdU incorporating nuclei with respect to the total number of nuclei. A minimum of 500 cells were analyzed per replica, and each sample has 3 replicas.

### 3.4. Scanning Electron Microscopy of HMVECS on Different Topographies

On the 7th day of culture, the samples were fixed with 2.5% glutaraldehyde (Fluka, Singapore). The cells were then dehydrated through graded ethanol solutions. A final dehydration in 100% ethanol solution was carried out three times for 5 min each before the samples were subjected to critical point drying (Balzers, Hudson, NH, USA, Critical Point Dryer 030). The samples were then gold coated by ion sputtering (JEOL, Japan, JFC 1600 Fine 270 Gold Coater, 90 s, 10 mA) before SEM examination (SEM, FEI, Japan, Quanta FEG 200, HV mode) at an accelerating voltage of 10 kV.

### 3.5. Data Analysis

For every experiment that was conducted, there were 3 replicas for each type of substrate topography. All data are presented as the mean ± standard deviation (SD), unless otherwise specified. One factor analysis of variance (ANOVA) with repeated measures and Bonferroni post-test correction were used to analyze the statistical significance where indicated. Two-way ANOVA tests with repeated measures were used to analyze the interaction effects between samples grown in medium B and samples grown in EGM-2MV. The significance level was set to be p = 0.05.

## 4. Conclusions

This study has demonstrated that HMVECs respond differentially to PDMS substrates patterned with pillars and wells topographies of dimensions in the micro- and nano-range. In terms of morphology, pillars enhanced the regularity of both cell shape and area. Positive and distinct staining of tight junction proteins at cell boundaries were observed in patterned substrates grown in EGM-2MV, indicating the successful formation of closed endothelial monolayers and maintenance of the barrier function. Increased expression of Na^+^/K^+^ ATPase and increase in microvilli density on the cell surface could also be observed for the monolayers grown on all patterned substrates, though the results appeared the strongest on the micropillars and microwells. This implies that the topographical systems tested, especially the microwells and the micropillars, can enhance expression of pump and tight junction proteins, which are necessary for the functions of the corneal endothelium. The data obtained demonstrates that the behavior and growth of HMVECs can be directed when cultured on patterned substrates. Overall, the HMVEC monolayers formed on micropillars and microwells in EGM-2MV score the highest in terms of corneal endothelium resemblance. These microtopographies in combination with the EGM-2MV medium can be considered for use to induce the HMVEC phenotype to be more like the corneal endothelium, with retained pump and barrier functionality. The mechanism involved in the contact guidance of the cells by the substrate topographies will be of interest for further studies.

## Supplementary Files

Supplementary File 1 Supplementary (PDF, 702 KB)
