# Supplementary material for: Cultivation of Human Microvascular Endothelial Cells on Topographical Substrates to Mimic the Human Corneal Endothelium
Source: J Funct Biomater. 2013 Mar 21;4(1):38–58. doi: 10.3390/jfb4010038 (PMC4030909; doi:10.3390/jfb4010038)

Supplementary Figure 1. Immunofluorescence images of laminin coating on (**A**) 1 µm pillars; (**B**) 1 µm wells; (**C**) 200 nm pillars; (**D**) 250 nm wells; (**E**) Unpatterned substrate; (**F**) Unpatterned substrate coated with only 1X Phosphate Buffered Saline (PBS, no laminin coating). The samples were coated overnight with 10µg/mL of lamnin and washed once with 1X PBS before incubating with Alexa Fluor 546 Carboxylic Acid, Succinimidyl Ester (Invitrogen) diluted 1:10000 overnight at 4 °C. All samples were washed twice with 1X PBS and mounted onto coverslips using ProLong Gold Antifade mounting medium (Invitrogen). The stained samples were viewed with an epifluorescent microscope (Leica DM IRB).

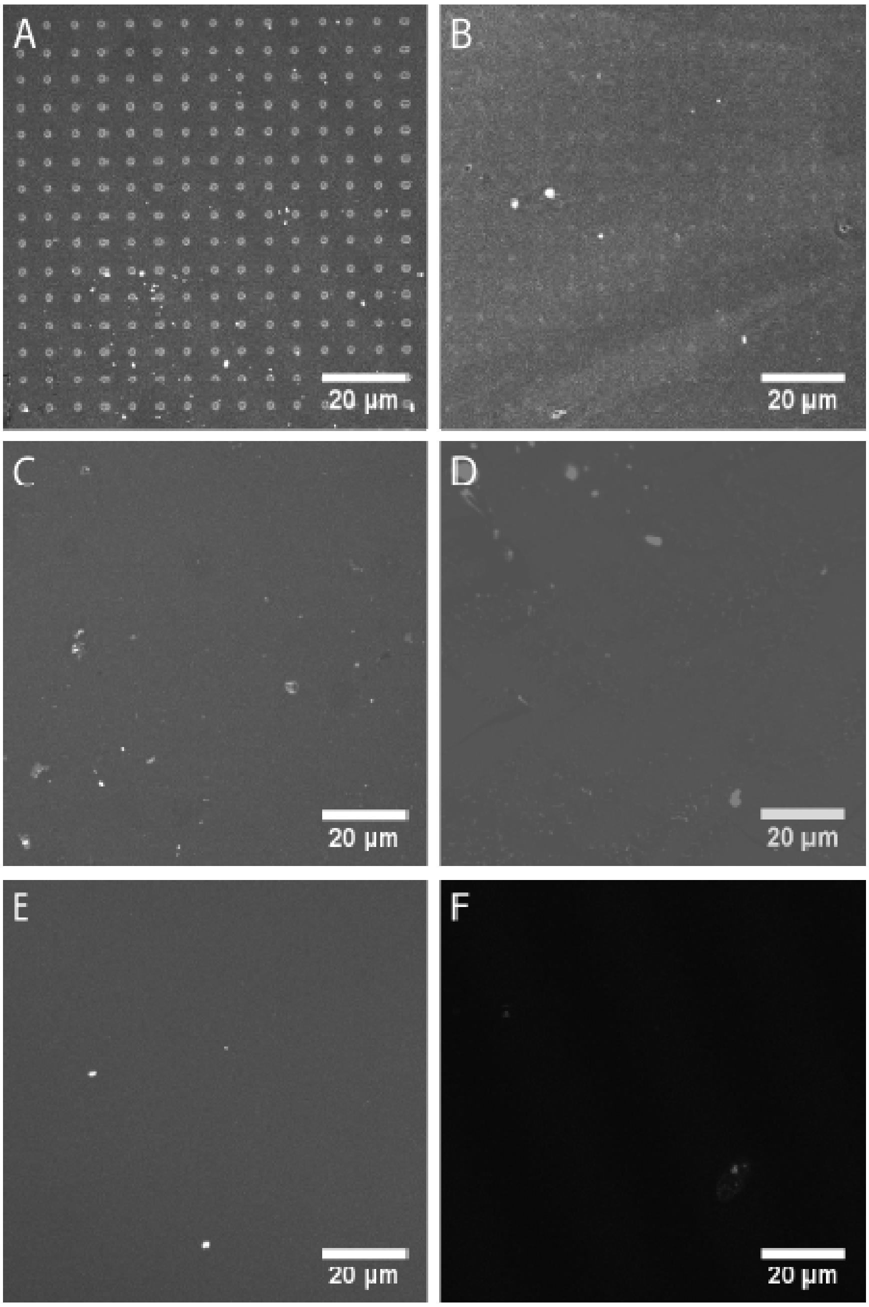

Supplementary Figure 2. Phase contrast images of (**A**) Human microvascular endothelial cells in culture flask; (**B**) Primary human corneal endothelial cells (HCEC); HCEC image was kindly provided by J. Mehta and G. Peh from the Singapore Eye Research Institute (SERI).

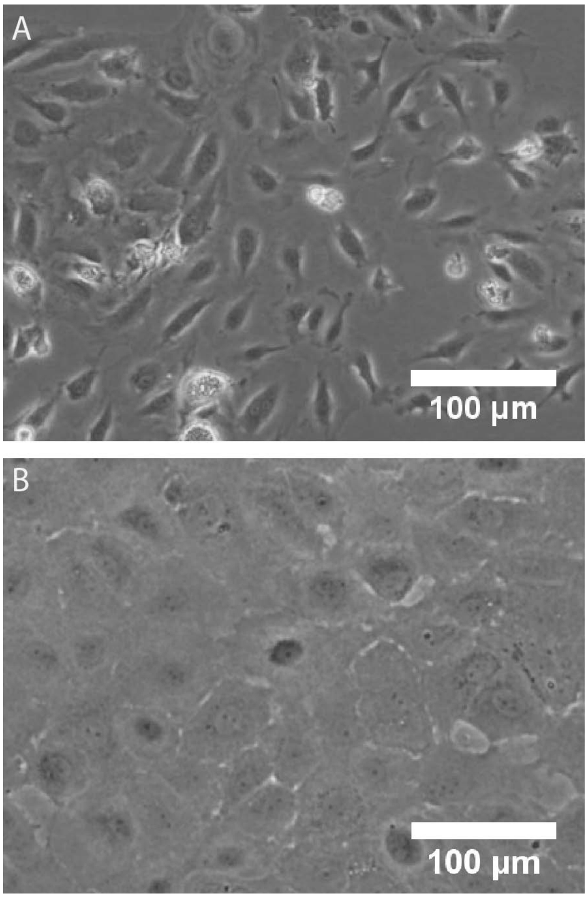

Supplement: Supplementary File 1 — Supplementary (PDF, 702 KB) [file jfb-04-00038-s001.pdf]
